# Ligustilide Attenuates Ischemia Reperfusion-Induced Hippocampal Neuronal Apoptosis *via* Activating the PI3K/Akt Pathway

**DOI:** 10.3389/fphar.2020.00979

**Published:** 2020-06-26

**Authors:** Qian Wu, Zhiguo Mao, Jiao Liu, Jinling Huang, Ning Wang

**Affiliations:** ^1^ Anhui Province Key Laboratory of Chinese Medicinal Formula, Anhui University of Chinese Medicine, Hefei, China; ^2^ College of Integrated Chinese and Western Medicine (College of Life Science), Anhui University of Chinese Medicine, Hefei, China; ^3^ Institute for Pharmacodynamics and Safety Evaluation of Chinese Medicine, Anhui Academy of Traditional Chinese Medicine, Hefei, China

**Keywords:** ligustilide, oxygen-glucose deprivation/reperfusion, hippocampal neurons, apoptosis, PI3K/Akt

## Abstract

Ligustilide (LIG), a main lipophilic component isolated from Cnidii Rhizoma (Cnidium officinale, rhizome) and Angelicae Gigantis Radix (Angelica gigas Nakai, root), has been shown to alleviate cerebral ischemia injury and paly a neuroprotective role. We investigated mechanisms underlying the antiapoptotic effects of LIG *in vitro* and *in vivo*, respectively, using cultured primary hippocampal neurons under oxygen-glucose deprivation/reperfusion (OGD/R) and rats under cerebral ischemia reperfusion(I/R) conditions. *In vitro* studies revealed that the suppressed apoptosis in hippocampal neurons upon LIG treatment was associated with reduced calcium influx and generation of reactive oxygen species. The LIG-treated hippocampal neurons exhibited decreased the ratio of Bax/Bcl-2, and the release of CytC from mitochondria as well as the expression of cleaved caspase-3, which were accompanied with enhanced the phosphorylation of Akt protein, in a PI3K-dependent manner. *In vivo* studies demonstrated a neuroprotective role of LIG in attenuating cerebral infarction volume, neurological injury and hippocampal neuron injury, suggesting that LIG could reverse ischemia reperfusion(I/R)-induced apoptosis of hippocampal neurons. These results together suggest that LIG may be considered as a neuroprotectant in the treatment of ischemia stroke.

## Introduction****

Ischemia stroke is the main subtype of stroke and accounts for approximately 80% of all patients, has become one of the leading cause of death and long-term disability worldwide, often resulting in irreversible brain damage and a loss of neuronal function (Feigin et al., 2014; Lapchak and Zhang, 2017). Tissue plasminogen activator (tPA) is currently the most effective clinical treatment for the treatment of ischemia stroke and approved by the Food and Drug Administration, but its use still remains limited because of its narrow therapeutic windows and reperfusion injury caused by restored blood flow (Henninger and Fisher, 2016; Sim et al., 2017; Yang S. H. et al., 2018). Self-repair is currently the most favorite potential therapy among researchers, oweing to benefits associated with enhancing the intrinsic ability of neurons.

As a form of regulated and programmed cell death, neuronal apoptosis plays an important role in cerebral ischemia-induced brain injury in both human and animal models (Sairanen et al., 2009). It’s not like the ischemic core, where most cells die through necrosis, apoptotic cells were mostly prevails in the penumbra. During the reperfusion phase, resupply of oxygen and nutrients would lead to a series of cellular damage due to intracellular Ca^2+^ accumulation, mitochondrial permeability transition pore (MPTP) opening and the increase of reactive oxygen species (ROS) generation (Dawson and Dawson, 2017), and subsequently increase the permeability of the mitochondrial membrane and induce the release of proapoptotic proteins such as Cytochrome C (CytC) or other apoptosis-inducing factor, which induces apoptosis (Kuida et al., 1998; Maes et al., 2017).Therefore, inhibiting the loss of hippocampal neurons provides a rationale against ischemia stroke. Hence, we have focused on the neuronal apoptosis regulation and possible mechanism in the penumbra after cerebral ischemia (Puyal et al., 2013; Aboutaleb et al., 2016).

**Figure 1:**
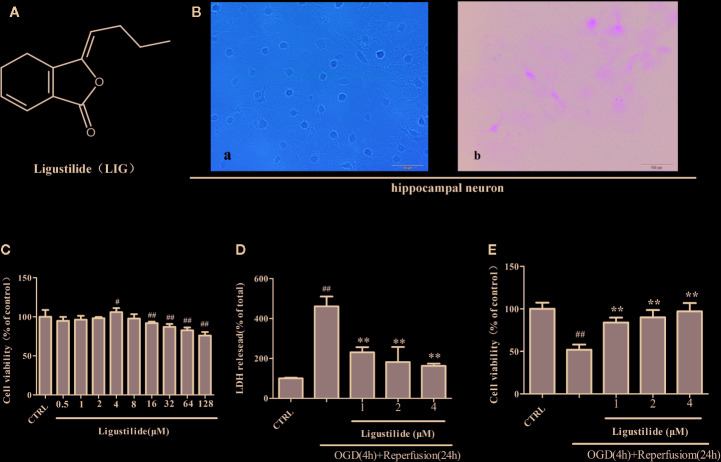
Ligustilide (LIG) promotes viability of hippocampal neurons induced by oxygen-glucose deprivation/reperfusion (OGD/R). **(A)** Chemical structure of LIG. B.Primary culture and identification of hippocampal neurons. **(B-a)** The seventh day of primary culture of hippocampal neuron by light microscope. **(B-b)** Hippocampal neuron were identified by MAP-2 immunostaining. **(C)** Hippocampal neurons were treated with LIG at various concentrations(0.5–128 μM) and the cell viability was measured by the MTT assay. **(D)** Effect of ligustilide on the release of LDH from hippocampal neurons damaged by OGD/R. **(E)** Cell viability was measured by the MTT assay after OGD/R and normal conditions. All data are expressed as means ± SD (n = 8). ^##^ *P* < 0.01 vs. control group; **P < 0.01 vs. group treated with OGD/R.

The rhizome Ligusticum Chuanxiong S.H.Qiu, Y.Q.Zeng, K.Y.Pan, Y.C.Tang & J.M.Xu (LC) is a Traditional Chinese Medicine known for its therapeutic effect on neurovascular and cardiovascular diseases, such as stroke, coronary heart disease, particularly in China, Japan and Korea. The essential oil from LC (EOLC) was suggested to be the therapeutic ingredients in this herb. Ligustilide (LIG; 3-butylidene-4,5-dihydrophthalide;chemical structure shown in **Figure 1A**), a major and high content of the EOLC, has been shown a widespread pharmacological activities, including neuroprotective (Xu et al., 2016), antioxidant (Donkor et al., 2016) and antiapoptotic (Chi et al., 2016) effects in ischemic stroke. *In vivo*, our previous work found that ligustilide could be detected in the cerebrospinal fluid of rats and ameliorates blood-brain barrier permeability (Li et al., 2017). Further experimental revealed that LIG had protective effect on glutamate-induced apoptosis in PC12 cells (Wu et al., 2015). However, mechanisms responsible for LIG’s neuroprotective effects have yet to be defined. In this study, we investigated whether ligustilide plays a protective role in brain through antiapoptosis pathway using primary hippocampal neurons and brain ischemia/reperfusion model.

## Materials and Methods

### Primary Culture and Identification of Hippocampal Neurons

All the use and care of animal procedures were in accordance with the National Institute of Health (NIH) guidelines for the Care and Use of Laboratory Animals, and approved by the Ethics Committee on Anhui University of Chinese Medicine. Primary culture of hippocampal neurons was performed according to relevant literature previously (Wang et al., 2003). Briefly, the dissected hippocampus from newborn rat was digested with trypsin (0.25%, 15 min, 37°C). Then the cells were seeded at a density of 5 × 10^5^ cells/ml onto poly-L-lysine (10 µg/ml, Sigma)-coated 96/6-well plates. Hippocampal neurons were grown in Neurobasal (Thermo, USA) medium supplemented with 2% B-27 (Thermo, USA) and 0.5mM glutamine (Gibco, USA), resulting in relatively pure primary neuronal cultures (~98%) as indicated by immunofluorescence for antimicrotubule-associated protein 2 (MAP-2, Abclonal, China). All hippocampal neurons were maintained for 7~8 days before subsequent experiments.

### OGD/R Model and LIG Treatment

LIG was bought from Chengdu Kemansite, Co, Ltd (Chengdu, China), stored at −20°C, and the purity of LIG was more than 98%. DMSO was used to dissolve LIG, and the final concentration of DMSO was less than 0.1% (vol/vol). LIG was diluted from 0.5 to 128 μM with neurobasal, and then added to hippocampal neurons 3h before OGD as pretreatment. The final concentration of DMSO was less than 0.1% (vol/vol).

For OGD treatment, hippocampal neurons were cultured in oxygen and glucose free DMEM. Then, hippocampal neurons were immediately placed in an anoxic incubator (Thermo, Waltham, MA) loaded with mixed gas containing 5% CO_2_ and 95% N_2_ for 2 h. For reperfusion, hippocampal neurons were replaced with normal medium and incubated under normal conditions at 37°C for 24 h for later experiments.

### MTT Assay

To explore the effect of LIG on hippocampal neurons viability, 3-(4,5-dimethylthiazol-2-yl)-2,5-diphenyl-tetrazolium-bromide (MTT, Sigma) assay was performed. After the determined treatment, MTT was added to each well for 4h at 37℃, and then dimethyl sulfoxide (DMSO) was added to each well of the plate to dissolve the formazan crystals. The percentage of cell viability was calculated as follows: Cell viability (%)=absorbance value of sample/absorbance value of control ×100.

### LDH Release Assay

The intracellular enzyme LDH will leaks into medium through the damaged cell membrane when cells are damaged. Cytotoxicity was evaluated with LDH activity assay kit (Nanjing Key-Gen Biotech, China). The medium was collected and centrifuged after each treatment, and the supernatant was taken for LDH detection. The activity was spectrophotometrically measured by a microplate reader at optical density of 340 nm. The results were expressed as percentage of control.

### Apoptosis Morphology Observation by Hochest 33258

Hippocampal neurons were pretreated with different concentrations of LIG before OGD/R. Following incubation, the hippocampal neurons were fixed with 4% polyoxymethylene for 30min, incubated with 10µg/ml Hochest 33258 (Beyotime Biotechnology, China) dye for 30 min at 37℃ and washed with PBS three times. Finally, images were taken using an Olympus microscope.

### Flow Cytometry Annexin V-FITC-PI Assay

The antiapoptotic effect of LIG was detected with Annexin V-FITC Apoptosis Detection Kit (Biouniquer Tech, China) according to the manufacturer’s protocol (Xiao et al., 2017). Briefly, hippocampal neurons were collected by centrifugation, twice washes with cold PBS and stained for 15 min with annexinV–fluorescein isothiocyanate (FITC) and propidium iodide (PI) in the buffer at room temperature in the dark. The apoptosis rate was analyzed by flow cytometry.

### Measurement of Intracellular ROS and Ca^2+^

Flow cytometry was used to analyze the generation of intracellular ROS and Ca^2+^ by the fluorescence probe 2′7′-dichlorodihydrofluorescein diacetate (DCFH-DA, Sigma) and Fluo-3/AM (Beyotime Biotechnology, China). After the cells were subjected to the determined treatment, 100 µM DCFH-DA and 5 µM Fluo-3/AM working solution was added into the medium and incubated for 30 min at 37°C. Cells were collected and washed with PBS and immediately analyzed.

### Western Blot Analysis

Protein expression was performed by Western blotting. After treatment, the supernatant was discarded. Hippocampal neurons were washed with cold PBS for twice and lysed in lysis buffer (Beyotime Biotechnology, China) containing 1mM phenylmethane sulfonyl fluoride (Beyotime Biotechnology, China) for 30 min on ice. The cells were collected and centrifuged at 12,000 rpm for 15 min at 4°C. The supernatant was collected and transferred to a new tube. The concentration of protein was determined using the BCA protein assay kit (Beyotime Biotechnology, China). The proteins were electrophoresed on a 12% (w/v) sulfate-polyacrylamide gel electrophoresis (SDS-PAGE) gel, transferred to a polyvinylidene difluoride (PVDF) membrane, which were then incubated with primary antibody against CytC, Bax, Bcl-2, Akt, p-Akt, cleaved caspase-3, and β-actin (1:1.000 dilution, Santa Cruz, USA) at 4°C overnight. Following twice wash with Tris-buffered saline with Tween 20 (TBST), the membrane was incubated with alkaline phosphatase-goat anti-rabbit IgG (1:1,000, Santa Cruz, USA) for 2 h at 37°C. The activity of peroxidase on the blot was developed using the enhanced chemiluminescence (ECL) detection reagents (Thermo, USA). Images were captured using an Amersham Imager 600 (General Electric, USA). β-actin was used as internal protein controls to represent the relative amount of the target protein.

### Cerebral I/R Model and LIG Treatment

Adult male Sprague Dawley (SD) rats (body weight, 250 ± 10 g) were provided by Jested Laboratory Animal Co., Ltd. Certificate number: (SCXK: Anhui, 2017-001). All experiments were approved by the Animal Care and Use Committee of Anhui University of Chinese Medicine and were in complete compliance with the National Institutes of Health Guide for the Care and Use of Laboratory Animals. Transient focal cerebral ischemia was induced by occlusion of the middle cerebral artery (MCAO) as previously described (Fan et al., 2011). Briefly, after the rats were anesthetized with 10% chloral hydrate (350 mg/kg) intraperitoneally, a 6-0 nylon monofilament suture was inserted 10 mm into the internal carotid to occlude the origin of the MCA. Animals with less than 80% reduction in CBF were excluded from the study. Body temperature was maintained at 37°C during surgery and for 2 h after the start of reperfusion. The experimental rats were randomly divided into three groups: sham operation group(sham group), cerebral ischemia-reperfusion group (model group) and ischemia-reperfusion+LIG group(LIG group). Rats in the sham group were only operated on in an identical manner except the occlusion. LIG (20 mg/kg/day, in 3% Tween 80) treated group were injected intraperitoneally at the onset after I/R insult and the sham group was injected with the equal volume of saline (3% Tween 80 alone), all for 7 days (n = 10 per group).

### Neurological Score

Neurological behavior was estimated at seventh day after MCAO according to the scales described by Longa (Fan et al., 2011). The neurological deficit was scored on a five-point scale. Score 0 = no neurologic deficit, score 1 = failure to extend forepaw fully, score 2 = circling to the left, score 3 = falling to the left or no spontaneous motor activity, and score 4 = do not walk spontaneously and experience a lowered stage of consciousness. The experimental results were obtained by the experimenter unaware of the experiment.

### Estimation of Infarction Volume

To measure infarct volume, brain slices were stained with 2% 2,3,5- triphenyltetrazolium (TTC, Sigma), at 2-mm intervals. Image Pro Plus 7.0 software was used to analyze and measure infracts volume.

### Ultrastructure Observation With Transmission Electron Microscopy

Two rats in each group were anesthetized and perfused with precooled PBS followed by 4% paraformaldehyde and 0.25% glutaraldehyde. The hippocampus was stripped from the brain and set in 4% paraformaldehyde solution, then cut into 50µm sections on a vibratome, embedded into 2.5% glutaraldehyde at 4°C. Put the chopped hippocampus in 2% osmium for 1.5 h, dehydrated, and embedded in Epon 812 Resin. Ultrathin sections (100 nm) were cut and stained with uranyl acetate and lead citrate, and then viewed under a transmission electron microscope (TEM).

### Statistical Analysis

Data are expressed as means ± SD. To compare means among three or more independent groups, one way ANOVA with multiple comparisons using Dunnett’s test were performed for statistical comparisons. For all tests, a value of P<0.05 was accepted as statistically significant.

## Results

### LIG Promotes Viability of Hippocampal Neurons after OGD/R-Induced Injury

We chose hippocampal neurons for our study because they are extremely sensitive to ischemia stroke. Rat hippocampal neurons were isolated and cultured, and then identified by immunofluorescence. At the seventh day, hippocampal neuron showed highly developed protrusions and interconnected, forming a dense network of nerve fibers **(** **Figure 1B-a** **)**. MAP-2, enriched in neuronal dendrites, is an intracellular neuronal specific marker. Immunofluorescence assay of MAP-2 showed that the bodies and dendrites of positive cells appear fluorescent green in most cells and purity of hippocampal neurons reached to 98% **(** **Figure 1B-b** **)**.

Hippocampal neurons were cultured with different concentrations (0.5–128 μM) of LIG for 24 h in order to screen the nontoxic dosage, and cell viability was detected by the MTT assay. Figure 1C shows that LIG exerted no cytotoxicity on hippocampal neurons at concentrations up to 8μM. To explore whether LIG could protect hippocampal neurons against OGD/R injury, we measured the cytotoxicity and viability by LDH assay and MTT assay. In **Figure 1D**, pretreatment with LIG (1, 2, and 4 μM) reduced remarkably the LDH release (230.81% ± 25.96%, 181.83% ± 75.75%, and 162.79% ± 11.34%, respectively) after OGD/R (461.40% ± 49.17%). In the same way, **Figure 1E** shows that the cell viability was reversed from 51.95% to 97.05% of control after OGD/R.

### LIG Inhibited OGD/R-Induced Ca^2+^ Influx and Oxidative Stress in Hippocampal Neurons

**Figure 2:**
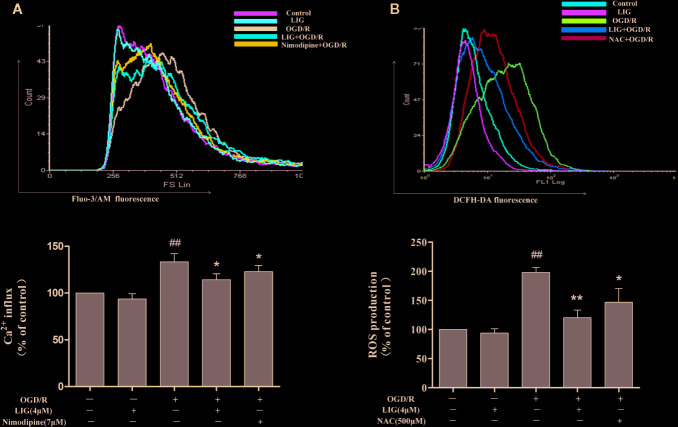
Ligustilide (LIG) attenuated Ca^2+^ influx and reactive oxygen species (ROS) levels in oxygen-glucose deprivation/reperfusion (OGD/R)-induced hippocampal neurons injury. (**A)** Effect of ligustilide on OGD/R-stimulated intracellular Ca^2+^ influx. **(B)** Effect of ligustilide on OGD/R-induced ROS overproduction. Cells were treated with LIG (1, 2, and 4 μM) for 3 h, and then subject to OGD/R. Fluo-3/AM and DCFH-DA were used to measure Ca^2+^ influx and ROS levels. The data are represented as means ± SD for three independent experiments. Each of the experiments was repeated three times. The data are means ± SD. ^##^ *P* < 0.01 vs. control group; *P < 0.05, **P < 0.01 vs. group treated with OGD/R.

Studies have indicated that cerebral ischemia could induced intracellular Ca^2+^ accumulation (Seidlmayer et al., 2015). Nimodipine is a calcium antagonist acting selectively on cerebrovascular smooth muscle and used for neuronal protection of ischemic in clinical. So we use it as a positive drug when we detect Intracellular Ca^2+^. **Figure 2A** shows that pretreatment with LIG obviously reduced the increased the accumulation of Ca^2+^ induced by OGD/R. The results indicated that LIG attenuated OGD/R-induced Ca^2+^ influx. During ischemia stroke, ROS play a key roles in many pathological processes. NAC is ROS scavenger, so we use it as a positive drug. In **Figure 2B**, the effect of LIG against OGD/R-induced oxidative stress was detected by Flow cytometry. The percentage of DCFH-DA fluorescence intensity was as an index of the rate of ROS production. While after exposure to OGD/R, ROS production dramatically increased compared with the control group. Nevertheless, pretreatment with LIG (1, 2, and 4 μM) obviously decreased OGD/R-induced ROS generation levels.

### LIG Inhibits OGD/R-Induced Apoptosis of Hippocampal Neurons.

**Figure 3:**
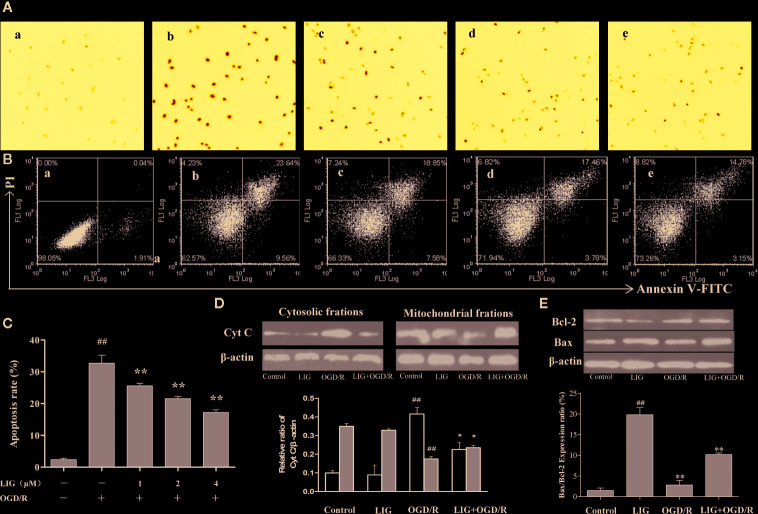
The effect of ligustilide on oxygen-glucose deprivation/reperfusion (OGD/R)-induced apoptosis of hippocampal neurons. **(A)** The percentage of hippocampal neurons apoptosis was analyzed by staining cells with Hoechst 8 (blue represents viable cells, ×200). **(B, C)** Apoptotic cells rate were detected by flow cytometry. (a) Control group; (b) OGD/R; (c) 1 μM LIG+OGD/R; (d) 2μM LIG+OGD/R; (e) 4 μM LIG+OGD/R. **(D)** Western blot image and the expression analysis of Cyt C in mitochondrial and cytosolic fractions. **(E)** Western blot image and the expression analysis of Bcl-2 and Bax. Data shown are the results of three different experiments. ^##^ *P* < 0.01 vs. control group; *P < 0.01, **P < 0.01 vs. group treated with OGD/R.

To explore the effect of LIG on OGD/R-induced apoptosis, we measured the cell apoptosis through hochest 33258 and flow cytometry. Hochest 33258 penetrates injured nucleus and labels the nuclei of living cells and of cells in the early stage of apoptosis. Under fluorescence microscopy, we found most hippocampal neurons in control group emitted weak blue fluorescent, while lots of neurons emitted strong blue fluorescence after OGD/R. However, a few cells emitted strong blue fluorescent in the pretreatment LIG (**Figure 3A**). To quantitatively examine apoptotic rate, Annexin V/PI double staining assay was used to detect cell apoptosis by FCM. After hippocampal neurons were exposed to OGD/R, the apoptosis ratio increased to 32.73%. Nevertheless, pretreatment with LIG (1, 2, and 4 μM) significantly attenuated cell apoptosis ratio to 25.59%, 21.54%, and 17.20%, respectively. These results suggested that LIG reduced OGD/R-induced apoptosis rate in hippocampal neurons (**Figures 3B, C**). Cyt C is mainly positioned on the mitochondria normally, whereas it will release from mitochondria to cytoplasm when damaged. **Figure 3D** indicated that the expression level of Cyt C under OGD/R attenuated obviously than the control group in the mitochondrial fraction of hippocampal neurons. However, LIG (4 μM) pretreatment obviously attenuated the increased level of Cyt C from the mitochondria to the cytoplasm induced by OGD/R. In addition, the ratio of Bax/Bcl-2 were significant increased under OGD/R compared to control group, while pretreatment with LIG (4 μM) reduced the ratio of Bax/Bcl-2 remarkably (**Figure 3E**), which are all apoptosis-related proteins. It verified LIG could inhibit apoptosis of hippocampal neurons induced by OGD/R.

### LIG Inhibited OGD/R-Induced Apoptosis by Activating the PI3K/Akt Signaling Pathway

**Figure 4:**
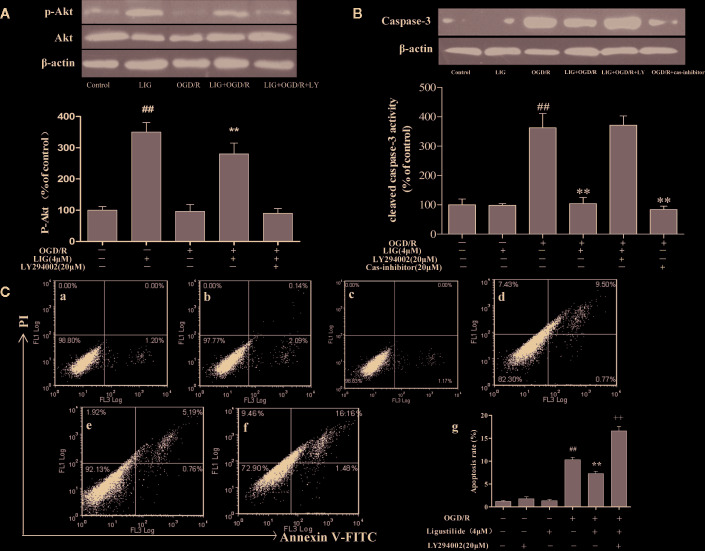
Role of PI3K/Akt in the antiapoptotic effect by ligustilide. **(A, B)** Western blot image and the expression analysis of P-Akt and Caspase-3. **(C)** Apoptotic cells rate were detected by flow cytometry. **(C-a)** Control group; **(C-b)** 20μM LY294002; **(C-c)** 4-μM LIG; **(C-d)** OGD/R; **(C-e)** 4 μM LIG+OGD/R; **(C-f)** 4-μM LIG+20μM LY294002+OGD/R. **(C-g)** The apoptotic cell rate of each group. Data represent the mean ± SD of three independent experiments.^##^ *P* < 0.01 *vs.* control group;**P < 0.01 vs. group treated with OGD/R; **^++^***P* < 0.01 vs. LIG+OGD/R group.

To determine whether the activation of PI3K/Akt pathway is involved in the neuroprotective effects of LIG, hippocampal neurons were pretreated with LY294002 (20µM), a PI3K inhibitor, 3 h before OGD/R. As shown in **Figure 4A**, the effect of LIG on the expression of phosphorylated Akt (p-Akt) were fully blocked by LY294002. To further elucidate the role of PI3K/Akt signaling pathway on apoptosis of hippocampal neurons following OGD/R, the expression of cleaved-caspase-3 was determined. As shown in **Figure 4B**, the activity of cleaved-caspase-3 was significantly increased under OGD/R, while it was decreased in the LIG-treated group. But this effect of antiapoptosis was weakened in the LY294002 treatment group.

The results of apoptosis rate detected by flow cytometry showed that pretreatment with LIG was remarkably lower (7.27%) compared to the OGD/R group (10.31%). Further research found that the apoptosis ratio of hippocampal neurons in the PI3K/Akt inhibitor group (20-µM LY294002 + 4-µM LIG + OGD/R) (16.59%) was significantly increased compared to the group pretreatment with LIG (**Figure 4C**). These result demonstrate that OGD/R-induced apoptosis was inhibited by LIG treatment, which was associated with activation of the PI3K/Akt signaling pathway.

### Effect of LIG on the Cerebral Infarct Volume and Neurological Deficits in Rats Induced by Cerebral Ischemia Injury

**Figure 5:**
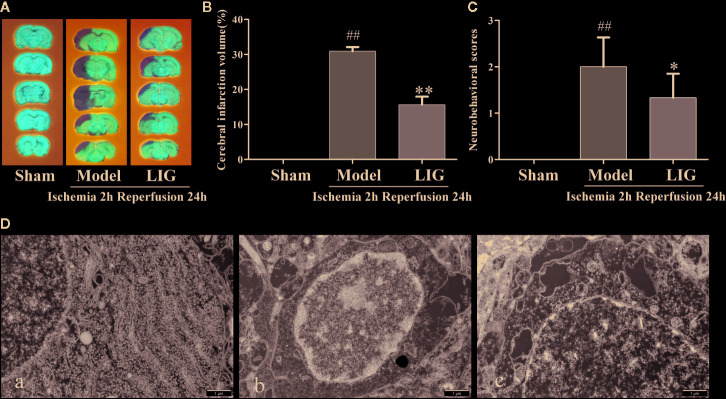
Effects of ligustilide(LIG) on cerebral infarct volume and neurological deficits in rats induced by cerebral ischemia injury. **(A)** Cerebral infarct volumes were determined by TTC staining. The pale area represents infarct area and red color stained area represents normal area. **(B)** Quantitative analysis of brain infarct volume. **(C)** Effect of LIG on neurological deficits in cerebral ischemia rats at the seventh day. Values are means ± SD (n = 10). **(D)** The postischemic ultrastructure of hippocampal neurons after LIG administration was examined by TEM (×20000). **(a)** Sham group; **(b)** Model group; **(c)** I/R+LIG (20 mg/kg) group. All data are expressed as mean ± SD, n = 10, ^##^ *p* < 0.01 vs. sham group, *p < 0.05, **p < 0.01 vs. model group.

Cerebral infraction was estimated by TTC staining after the day of occlusion of artery. As shown in **Figure 5A**. Compare with the sham group, the infract volume in the model group were increased after occlusion of carotid artery. However, the infarct volume was obviously reduced in I/R+LIG (20 mg/kg) group (**Figure 5B** **)**. The results showed that LIG could reduce cerebral edema caused by I/R. And then we measure the neurological deficit on the basis of scoring scale of change in neurological behavior. In the I/R group, neurological scores were significantly increased compared to sham group as the days increases from 1 to 7 days. Whereas, LIG (20 mg/kg/day) treatment obviously reduced the neurological score (**Figure 5C**). We also observed the ultrastructural of ischemic neurons. In sham group, the chromatin of hippocampal neuron was evenly distributed without dissolving and pyknosis, the morphology of the nucleus and organelles was regular (**Figure 5D-a**).While the hippocampus neurons of rats were seriously damaged, cell body was shrunk, cytoplasm was condensed, karyopyknosis dissolving phenomenon occurred, the mitochondrial cristae fracture was broken, and the apoptotic bodies occurred in the I/R group (**Figure 5D-b**). However, pretreatment with LIG showed that chromatin was condensed slightly, the nucleoli and it’s membrane remain intact (**Figure 5D-c**). These results demonstrated LIG could attenuate I/R-induced injury of hippocampal neurons.

## Discussion

Stroke, the leading cause of adult disability and morbidity, remains the major cause of mortality in most developing and developed countries. The global economic costs and burden associated with stroke are high (Dawson and Dawson, 2017; Yang J. L. et al., 2018), rising from 38 million disability-adjusted life years (DALYs) in 1990 to 61 million DALYs in 2020. (*The Atlas of Heart Disease and Stroke from* http://www.who.int/cardiovascular_diseases/resources/atlas/en/ *)*. A large majority (80%–90%) of stroke cases are caused by obstruction of one or more cerebral arteries (Lapchak and Zhang, 2017). A lack of blood supply to cerebral regions disturbs cellular homeostasis, culminating in neuronal death. Apoptosis is the dominating way of cell death in ischemia penumbra (Aboutaleb et al., 2016), which can be initiated by disrupting mitochondria function and homeostasis, and releasing of pro-apoptotic proteins such as CytC or other apoptosis-inducing factor such as effector caspases and the final execution of apoptotic death (Galluzzi et al., 2012; Murphy and Hartley, 2018). We found that LIG treatment resulted in a significantly down-regulation the ratio of Bax/Bcl-2, the expression of CytC and cleaved caspase-3. These data together suggest strongly a neuroprotective function of LIG treatment on OGD/R-induced injury, and provide evidences for an antiapoptotic role of LIG.

PI3K/Akt pathway is a key survival promoting and antiapoptotic signaling pathway (Zhang et al., 2014; Ho et al., 2015). Activated Akt can regulate cell function by a cascade of downstream phosphorylation and interaction among target proteins, which, in turn, regulate many cellular activities and biological function in cell survival, apoptosis, proliferation, cytoskeleton change, carbohydrate metabolism, gene transcription, angiogenesis and other activities (Aksamitiene et al., 2012). After brain injury, multiple neurotrophic factors play a protective role in the brain by activating the PI3K/Akt signaling pathway (Kilic et al., 2017). It was also revealed that the activation of PI3K-Akt is mainly contributed to the neuroprotective effect through the post conditioning treatment (Lang and Ling, 2012). Many studies have demonstrated that PI3K/Akt signaling pathway plays an key role in the neuroprotective effect and neurofunctional repair against cerebral ischemia (Huang et al., 2014). Interestingly, we found that activation of PI3K/Akt signaling pathways upon LIG treatment is associated with inhibition of apoptosis in cultured primary hippocampal neuronals.

Natural compounds has been pointed to display well its neuroprotective effects both *in vivo* and *in vitro*. For instance, LIG is a lipophilic constituent of Ligusticum chuan xiong, that can quickly cross the blood-brain barrier (Shi et al., 2015). LIG shows various pharmacological effects, including neuroprotective (Xu et al., 2016), antioxidant effects (Donkor et al., 2016), antiapoptotic (Chi et al., 2016), antiinflammatory (Choi et al., 2018) and so on. (Kuang et al., 2014) reported that LIG has a remarkable neuroprotective effects on several central nervous system pathologies including inhibiting the proinflammatory mediators from microglia and astrocytes, which play vital role inneuronal. Another previous study indicated that the antiapoptotic effect of LIG on brain ischemia *in vivo* and in PC12 cells (Kuang et al., 2006; Yu et al., 2008). Our previous studies have shown that ligustilide could be detected in the cerebrospinal fluid of rats by HPLC and ameliorates the permeability of the blood-brain barrier *in vitro* (Li et al., 2017). In the present study, we found that LIG could significantly attenuate the oxygen-glucose deprivation/reperfusion-induced injury in hippocampal neurons. Further experiments showed that, LIG significantly alleviated ROS production and overloading calcium. In addition, the present study showed that LIG significantly suppressing the release of CytC and attenuates the expression of caspase-3 and increased Bcl-2 expression. Many studies have shown that PI3K-Akt signaling plays an important role in inhibiting apoptosis, thus protecting the nerve cell. Previous studies have suggested that the neuroprotective effect of LIG has been correlated to PI3K/Akt in PC12 cells in previous studies(Kuang et al., 2006; Yu et al., 2008; Qi et al., 2012). The present study further confirmed the neuroprotective effect of LIG and investigated the mechanism of LIG-mediated antiapoptotic effect against ischemia reperfusion-induced injury of hippocampal neurons. Our results show that there was no significant effect on the overall expression of Akt under OGD/R in the hippocampal neurons, while significantly reduced the p-Akt. LIG significantly up-regulated the expression of p-Akt in cells with OGD/R injury compared with the normal control group. As expected, when the activity of PI3K was inhibited by LY294002, attenuated LIG induced p-Akt.

To further verify whether the PI3K/Akt pathway activated by LIG is involved in the neuroprotective effect, we detected the expression of apoptotic protein cleaved caspase-3 and the apoptosis rate of hippocampal neurons. We found that the significantly increased of cleaved-caspase-3 under OGD/R was decreased in the LIG-treated group. But this effect of antiapoptosis was weakened in the LY294002 treatment group. Also, the results of apoptosis ratio by flow cytometry showed that pretreatment with LIG was remarkably lower (7.27%) than that in the OGD/R-treated group (10.31%). While the apoptosis ratio of hippocampal neurons were significantly increased in the PI3K/Akt inhibitor group (20-µM LY294002 +4-µM LIG +OGD/R) (16.59%). Our results suggest that the antiapoptotic effect of LIG might be related to the activation of PI3K/Akt pathway. Our experiments further confirmed the neuroprotective effect of LIG by attenuating cerebral infarction volume, neurological injury, and hippocampal neuron injury.

**Figure 6:**
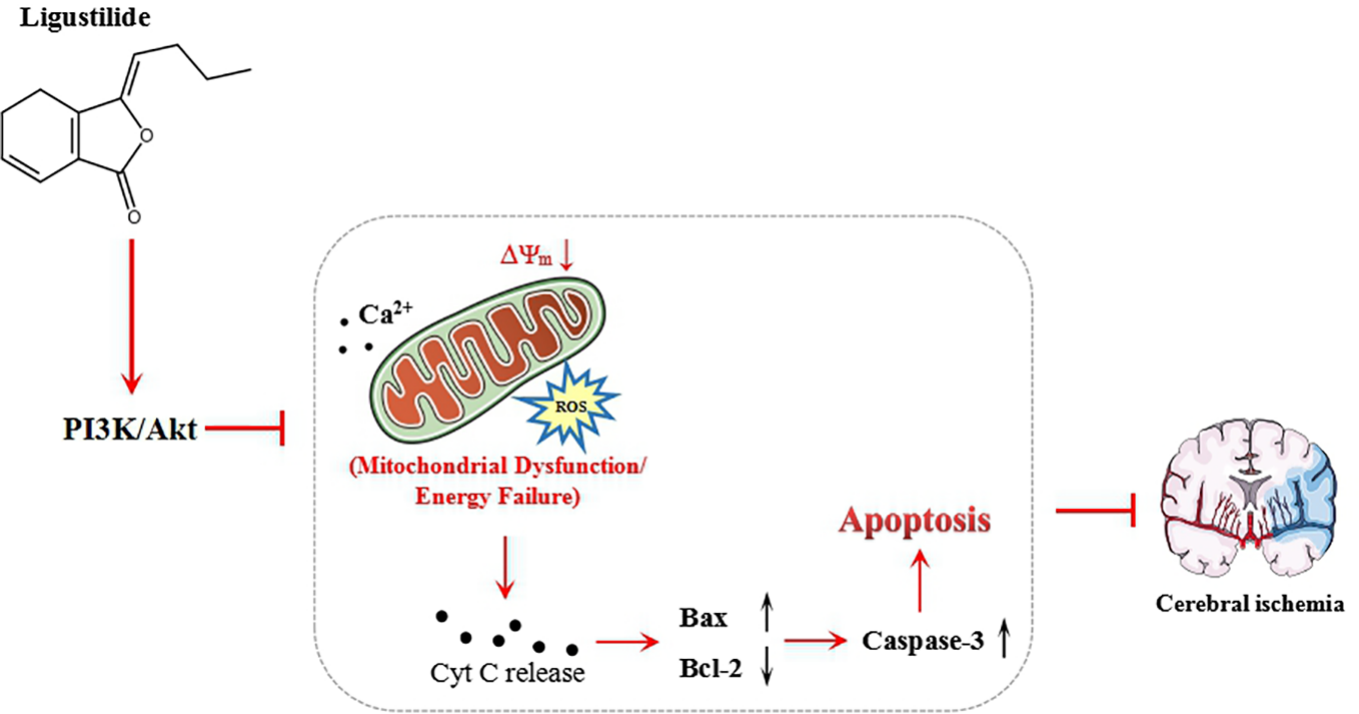
Schematic showing the mechanisms of ligustilide reduce the cell apoptosis injured by oxygen-glucose deprivation/reperfusion (OGD/R).

In summary, this study has demonstrated that LIG treatment can protect hippocampal neurons against OGD/R-induced injury by mitigating intracellular ROS, reducing calcium overload, and then attenuating apoptosis. The LIG-mediated antiapoptotic effect may be achieved through activation of PI3K/Akt signaling pathway (**Figure 6**). Our findings indicate that LIG is a promising neuroprotective agent in the treatment of ischemia stroke. However, whether LIG can be used in clinical treatment of cerebral infarction still requires further *in vivo* investigations to ascertain the therapeutic potential of ligustrazine in the treatment of ischemia stroke.

## Data Availability Statement

All datasets presented in this study are included in the article/supplementary material.

## Ethics Statement

The animal study was reviewed and approved by Animal Care and Use Committee of Anhui University of Chinese Medicine.

## Author Contributions

Study design: QW and NW. Data collection and analysis: QW, ZM, and JL. Study supervision: NW. Paper revising: QW, JH, and NW. All authors contributed to the article and approved the submitted version.

## Conflict of Interest

The authors declare that the research was conducted in the absence of any commercial or financial relationships that could be construed as a potential conflict of interest.
